# Liberale Leitlinien für eine deutsche Außen‑, Sicherheits- und Entwicklungspolitik aus einem Guss

**DOI:** 10.1007/s12399-021-00850-z

**Published:** 2021-05-17

**Authors:** Alexander Graf Lambsdorff

**Affiliations:** Fraktion der Freien Demokraten im Deutschen Bundestag, Platz der Republik 1, 11011 Berlin, Deutschland

**Keywords:** Deutsche Außenpolitik, Sicherheitspolitik, FDP, Freie Demokraten, Bundestagswahl 2021, German foreign policy, Security policy, FDP, Free Democratic Party, Elections

## Abstract

Die Covid-Pandemie verstärkt globale Entwicklungen, die bereits zuvor ihre Schatten voraus warfen. Eine neue Ära der Großmachtkonflikte bricht an, die Demokratie gerät weltweit in die Defensive. Insbesondere der Aufstieg Chinas fordert die Staatengemeinschaft heraus. Internationale Organisationen stehen unter Druck, während Herausforderungen wie der Klimawandel multilaterale Antworten erfordern. Deutschland wird daher international mehr Verantwortung übernehmen müssen. Der Beitrag stellt vor diesem Hintergrund die außen- und sicherheitspolitischen Positionen der Fraktion der Freien Demokraten vor.

## Abstract (en)

The COVID-19 pandemic is intensifying global developments that had already been looming prior to its outbreak. A new era of great power conflicts dawns; democracy has been put on the defensive. In particular, China’s rise has challenged the international community. International organisations are under pressure, while challenges like climate change require multilateral responses. Germany will therefore need to take on more responsibility internationally. Against this backdrop, the article introduces the foreign and security policy positions of the Parliamentary Group of the Free Democrats in the German Bundestag.

## Für Freiheit und Menschenrechte weltweit!

Die internationale Staatenwelt steht im kommenden Jahrzehnt vor tektonischen Verschiebungen. Besonders der Aufstieg Chinas fordert die EU und ihre demokratischen Partner wirtschaftlich, technologisch, gesellschaftlich und geopolitisch heraus. Der geopolitische Konflikt zwischen den USA und China im pazifischen Raum wird zur bestimmenden Machtauseinandersetzung in der internationalen Politik und wird auch für Deutschland und die EU weitreichende Folgen haben. Zugleich sehen wir, wie durch das Erstarken autoritärer Staaten Demokratie, Freiheit, Menschenrechte und multilaterale Kooperation weltweit in die Defensive geraten. Russland und China verfolgen unilateral und teilweise aggressiv ihre Interessen, auch gegenüber den eigenen Bürger*innen. Ihnen geht es nicht um Win-Win für möglichst viele, sondern um Macht und Geländegewinne. Gleichzeitig hat der Rückzug der USA als Garant der liberalen Weltordnung während der vier Jahre von Donald Trump als US-Präsident alte Gewissheiten in Deutschland und Europa erschüttert. Und trotz erster hoffnungsvoller Signale an die westlichen Verbündeten wird die neue US-Regierung unter Joe Biden nicht einfach zu einem status quo ante in der Außen- und Sicherheitspolitik zurückkehren können.

Das bedeutet: Deutschland und die Europäische Union müssen international in einer neuen Ära der Großmachtkonflikte bestehen. Gleichzeitig steht die EU mit der Bewältigung der Folgen der Covid-Pandemie vor ihrer größten Bewährungsprobe. Unser *European Way of Life* steht auf dem Spiel, wenn es nicht gelingt, den wirtschaftlichen Wiederaufschwung nach der Pandemie kraftvoll zu gestalten. Dafür muss die EU aber auch an sich selbst arbeiten. Sie muss sich der Erosion ihrer Rechtsstaatlichkeit in einzelnen Mitgliedstaaten entgegenstellen und sich nach innen wie nach außen als handlungsfähig beweisen. Aufgaben, Arbeitsweise und Zuständigkeiten müssen daher mutiger als in der Vergangenheit reformiert werden. Deutschland sollte als EU-Mitgliedstaat in Zukunft auch noch mehr Verantwortung international übernehmen und neue Ideen entwickeln.

Wir Freie Demokraten sind überzeugt, dass der diplomatische Einsatz für Freiheit, Menschenrechte, Demokratie und Rechtstaatlichkeit immer Grundpfeiler einer erfolgreichen deutschen Außenpolitik sein muss, die nicht nur unseren Interessen, sondern auch unseren Werten verpflichtet ist. Aus diesem Grunde haben wir als Partei und Fraktion unsere internationale Politik unter das Leitbild „Freiheit und Menschenrechte weltweit“ gestellt. Wir wollen uns gemeinsam mit unseren europäischen Partnern für die Bewahrung unserer freiheitlichen Lebensweise in Europa und den Schutz von Frieden und Menschenrechten international einsetzen. Die Stärkung der regelbasierten liberalen Weltordnung ist für uns zentral. Deshalb sind der Erhalt von handlungsfähigen internationalen Institutionen, die Stärkung einer freien und regelbasierten Welthandelsordnung, die Wiederbelebung der Abrüstung und Rüstungskontrolle von Massenvernichtungswaffen sowie die Durchsetzung von internationalem Recht Kernanliegen unserer liberalen Außen- und Sicherheitspolitik.

### Globalen Herausforderungen multilateral und vernetzt begegnen

In der Welt des 21. Jahrhunderts sind Chancen und Risiken auf gleiche Weise vernetzt, multidimensional und transnational. Das gilt für Herausforderungen wie den Klimawandel und Pandemien ebenso wie für die Nichtverbreitung von Massenvernichtungswaffen, die Abwehr von Cyberangriffen oder die Bekämpfung des internationalen Terrorismus. Unsere Antworten auf diese Herausforderungen müssen es ebenso sein – sowohl in der Zusammenarbeit mit unseren europäischen und internationalen Partnern, als auch in der Ausrichtung unseres eigenen internationalen Handelns als Bundesrepublik Deutschland.

### Für eine deutsche Außen‑, Sicherheits- und Entwicklungspolitik aus einem Guss

Die größte Herausforderung für die deutsche Außen‑, Sicherheits- und Entwicklungspolitik ist es vor dem Hintergrund dieser Entwicklungen, sich sowohl strategisch, institutionell und finanziell neu und besser aufzustellen. Wir müssen schneller diplomatisch die Initiative ergreifen, unser internationales Handeln vernetzter denken, uns besser mit unseren europäischen und internationalen Partnern abstimmen und wir müssen auch bereit sein, dass die Bundeswehr einen Beitrag leistet, wenn es erforderlich ist. Bundeskanzlerin Dr. Angela Merkel hat in ihrer Rede zum Bundeshaushalt 2020 betont, dass es von ganz besonderer Bedeutung sei, einen gemeinsamen Ansatz zu sehen, der sich nicht auf das Militärische konzentriere; das Eigentliche seien vielmehr die politischen Lösungen und die Entwicklungszusammenarbeit. Dieser Befund ist grundsätzlich richtig. Aber statt eines zeitgemäßen vernetzten Ansatzes, der die Ressorts und deren Instrumente wirkungsvoll koordiniert, ist das internationale Handeln der Großen Koalition geprägt durch Ressortdenken und eitle Rivalitäten zwischen den zuständigen Minister*innen. Unsere Partner erwarten aber von uns, dass Deutschland nicht passiv am Spielfeldrand steht, während andere den Takt vorgeben, die Ordnung der Welt neu sortieren und damit auch über Deutschlands und Europas Zukunft entscheiden.

Es ist deshalb höchste Zeit, dass Deutschland seine Außen‑, Sicherheits- und Entwicklungspolitik strategisch ausrichtet. Die Bundesrepublik braucht eine Außen‑, Sicherheits- und Entwicklungspolitik aus einem Guss, die verantwortungsbewusst und kohärent unsere Werte und Interessen im Verbund mit unseren Partnern und Verbündeten in EU und NATO wirksam umsetzt. Hierfür müssen die politischen Entscheidungsstrukturen angepasst und die finanziellen Voraussetzungen geschaffen werden. In einem ersten Schritt ist es notwendig, einen Nationalen Sicherheitsrat einzurichten. Er würde es ermöglichen, bei internationalen Herausforderungen vorausschauender sowie schneller planen, entscheiden und handeln zu können. Durch ein solches Gremium könnten Verantwortungsdiffusion und Blockaden zwischen den Ministerien und die damit verbundene Handlungsunfähigkeit überwunden werden. Zweitens braucht Deutschland eine politische Gesamtstrategie, die die Ziele und Prioritäten unserer Außen‑, Verteidigungs- und Entwicklungspolitik festlegt. Jede neue Bundesregierung sollte ein Jahr nach Amtsantritt ihre Gesamtstrategie dem Deutschen Bundestag vorlegen. Auf diese Weise würde nicht nur eine gemeinsame Entscheidungsfindung forciert, sondern auch die Transparenz der Entscheidungen erhöht werden. Drittens schließlich sollte Deutschland im Sinne eines vernetzten Ansatzes 3 % seines Bruttoinlandsprodukts in internationale Sicherheit (3D – *defence, development and diplomacy*) investieren und so seine in der NATO eingegangenen Verpflichtungen erfüllen, seine Entwicklungspolitik verstetigen und seine Diplomatie stärken.

## Für die Selbstbehauptung Europas in einer unruhigen Welt

Der Gedanke eines *vereinten Europas* ist bereits in der Präambel des Grundgesetzes verankert, genauso der Anspruch der Mütter und Väter des Grundgesetzes an die Bundesrepublik, in diesem vereinten Europa dem Frieden der Welt zu dienen. Die Europäische Union ist eines der größten Friedensprojekte der Geschichte. Als Fraktion der Freien Demokraten sind wir überzeugte Europäer*innen. Wir wollen nicht hinnehmen, dass die EU zur Projektionsfläche für Globalisierungsängste und als Spielball von Populist*innen genutzt wird. Deutschland sollte deshalb die Initiative übernehmen, um Erosionstendenzen in der EU entgegenzuwirken. In einzelnen EU-Mitgliedsstaaten schreitet die Einschränkung von Freiheitsrechten, Demokratie und Rechtsstaatlichkeit immer stärker voran. Laut den Freedom-House-Länderberichten haben mehrere Mitgliedstaaten der EU in den vergangenen Jahren eine Verschlechterung ihres Gesamtwertes für politische Rechte und bürgerliche Freiheiten erfahren, davon vier in erheblichem Maße.^1^ Auch das Europäische Parlament hat sich wiederholt kritisch zur Lage der Rechtsstaatlichkeit und der Grundrechte in Teilen der EU geäußert, während Mitgliedstaaten und Kommission immer noch zu zurückhaltend bleiben. Europa braucht einen Rettungsschirm für den Rechtsstaat durch eine europäische Grundwerteinitiative, um den Schutz von Rechtstaatlichkeit, Menschen- und Bürgerrechten zu verbessern. Hierzu müssen wir insbesondere die Möglichkeit schaffen, dass bei Verstößen gegen das Rechtsstaatsprinzip die Zahlungen von EU-Mitteln ausgesetzt werden kann.

Zudem ist es notwendig, die EU durch institutionelle Reformen zu stärken. Nach einem erfolgreichen Abschluss der Konferenz zur Zukunft Europas sollten die EU-Mitgliedstaaten einen Verfassungskonvent einberufen. Ziel des Konvents muss es sein, der Union eine rechtsverbindliche Verfassung mit einem Grundrechtekatalog und starken Institutionen zu geben. Die endgültige Entscheidung über eine Europäische Verfassung sollte bei den EU-Bürger*innen liegen, die in einer gemeinsamen europäischen Volksabstimmung entscheiden und damit die Grundlage für einen föderal und dezentral verfassten Europäischen Bundesstaat schaffen. Bis der große Schritt zu einer europäischen Verfassung erfolgreich gegangen wurde, sollte aus unserer Sicht die europäische Integration durch ein „Europa der verschiedenen Geschwindigkeiten“ vertieft werden.

Der fortschreitende Wandel der Weltordnung und konkrete Umwälzungen in unmittelbarer Nachbarschaft der EU erfordern es, dass Europa schneller und besser handlungsfähig ist. Die EU wird auf der Weltbühne nur gehört werden, wenn sie mit einer Stimme spricht. Die EU braucht deshalb eine Gemeinsame Außen- und Sicherheitspolitik (GASP), die den Namen auch verdient. Die Einstimmigkeit sollte im EU-Ministerrat in die qualifizierte Mehrheit überführt werden. Der Europäische Auswärtige Dienst (EAD) muss gestärkt und der Hohe Vertreter der EU für Außen- und Sicherheitspolitik so befähigt werden, dass er zukünftig als vollwertiger *EU-Außenminister* agieren kann. Gemeinsames Handeln wird so zur Regel und nationalstaatliche Alleingänge zur Ausnahme. In der Gemeinsamen Sicherheits- und Verteidigungspolitik (GSVP) sollte das langfristige Ziel der Aufbau einer europäischen Armee unter gemeinsamem Oberbefehl und unter parlamentarischer Kontrolle sein. Dazu ist eine schrittweise engere Verzahnung und der Ausbau gemeinsamer Fähigkeiten der Streitkräfte der integrationswilligen EU-Mitgliedsländer nötig.

## Multilateralismus aus der Krise führen, Handlungsfähigkeit von internationalen Organisationen sicherstellen

### Die Vereinten Nationen stärken

Die Vereinten Nationen spielen für die Stärkung des Multilateralismus eine Schlüsselrolle. 75 Jahre nach ihrer Gründung ist es aber unübersehbar, dass sich die Welt weiterentwickelt hat. Der Sicherheitsrat und viele weitere UN-Gremien haben dieser Entwicklung nur bedingt Rechnung getragen. Auch wenn die Zahl der nichtständigen Mitglieder im Sicherheitsrat in der Zwischenzeit von sechs auf zehn gewachsen ist, spiegelt dieser Aufwuchs bei weitem nicht die Zunahme an Mitgliedern in der gleichen Zeit wider. Um die Akzeptanz und Arbeitsfähigkeit des Sicherheitsrates zu erhöhen, sind Reformen dringend notwendig. Die Fraktion der Freien Demokraten setzt sich deshalb für einen ständigen europäischen Sitz im Sicherheitsrat der Vereinten Nationen ein. Darüber hinaus wird die Handlungsfähigkeit der UN durch mangelnde finanzielle Unterstützung sowie durch zweckgebundene Zahlungen bedroht. Durch die Zweckgebundenheit gehen Gelder zwar formal an eine internationale Organisation, in der Praxis sind sie aber der multilateralen Entscheidungsfindung zur gemeinschaftlicher Lösung globaler Herausforderungen entzogen. Die UN-Sonderorganisationen müssen deshalb endlich auf eine solide finanzielle Grundlage gestellt werden. Wir werben dafür, dass internationale Geldgeber einen höheren Anteil ihrer Beiträge dem ordentlichen Haushalt als frei verfügbare Mittel zur Verfügung stellen, um die Praxis verspäteter Zuschüsse als gebundene Projektmittel zu überwinden. Dies gilt insbesondere für Welternährungsprogramm (WFP), Bevölkerungsfonds (UNFPA), Kinderhilfswerk (UNICEF), Entwicklungsprogramm (UNDP), Umweltprogramm (UNEP), Weltgesundheitsorganisation (WHO), UN-Habitat sowie den Hochkommissaren für Flüchtlinge (UNHCR) und für Menschenrechte (OHCHR).

### Klares Bekenntnis zur NATO

Über 70 Jahre nach ihrer Gründung ist die NATO noch immer ein konkurrenzlos erfolgreiches Sicherheitsbündnis. Deutschland sollte sich dafür einsetzen, dass die Verteidigungsallianz auch in Zukunft Garant für unsere Sicherheit sein kann. Hierzu müssen die zentralen Beschlüsse der Allianz von Wales und Warschau in vollem Umfang umgesetzt werden. Insgesamt ist es notwendig, das Atlantische Bündnis politisch-strategisch sowie militärisch weiterzuentwickeln. Der Reflexionsprozess war hierfür ein wichtiger erster Schritt. Die NATO-Mitgliedsstaaten sollten aber darüber hinaus den NATO-Generalsekretär mit der Erarbeitung eines neuen Strategischen Konzepts beauftragen. Das letzte große Grundlagendokument des Bündnisses stammt aus dem Jahr 2010. Seine damaligen Schwerpunkte – kollektive Verteidigung, gemeinsames Krisenmanagement und kooperative Sicherheit sind weiterhin gültig. Spätestens mit der völkerrechtswidrigen Annexion der Krim durch Russland hat sich die Bedrohungslage jedoch entscheidend verändert. Auch der Aufstieg Chinas hat sicherheitspolitische Auswirkungen auf die NATO. Es braucht deshalb jetzt eine große, gemeinsame Kraftanstrengung, um Klarheit über Prioritäten und Ziele der NATO für das kommende Jahrzehnt zu definieren und insbesondere eine Strategie für den Umgang mit China zu entwickeln. Es ist zudem notwendig, den europäischen Pfeiler in der NATO zu stärken und dadurch auch die Handlungsfähigkeit der EU zu erhöhen. Nur ein komplementäres Handeln von NATO und EU erlaubt es, auf die heutigen und künftigen sicherheitspolitischen Herausforderungen angemessen reagieren zu können.

## Mehr Verantwortung wagen – liberale Eckpunkte für eine deutsche Außen- und Sicherheitspolitik gegenüber den USA, Russland und China

### Transatlantische Beziehungen erneuern

Die Wahl von Joe Biden zum nächsten Präsidenten der Vereinigten Staaten von Amerika ist die große Chance, die transatlantischen Beziehungen für das kommende Jahrzehnt zu erneuern; Deutschland und die EU müssen diese Chance nutzen. Deutschland braucht hierzu eine Amerika-Strategie. Der Atlantik ist spürbar breiter geworden unter Trump. Doch in einer Zeit, in der autoritäre Staaten erstarken und die Demokratie weltweit in die Defensive gerät, bedarf es einer besonderen Kraftanstrengung, um die westliche Staatengemeinschaft zusammenzuhalten. Echte Antworten auf globale Herausforderungen wie Klimawandel, Atomwaffenkontrolle oder Pandemien erfordern die USA am Verhandlungstisch – auch das ist eine Lehre der Trump-Jahre. Zwar ist das Engagement der USA in internationalen Organisationen und als Garant für die liberale Weltordnung auch in ihrem eigenen Interesse. Joe Biden hat als eine seiner ersten Amtshandlungen entschieden, dass die USA wieder dem Pariser Klimaabkommen beitreten und in die Weltgesundheitsorganisation zurückkehren. Das begrüßen wir ausdrücklich, doch wissen wir auch, dass die Biden-Administration den außenpolitischen Kurs der USA der letzten vier Jahre nicht durch einen Druck auf den Reset-Knopf ungeschehen machen kann. Deutschland und die EU müssen daher die teilweise berechtigte Kritik der USA an der Funktions- und Handlungsfähigkeit internationaler Organisationen und multilateraler Kooperationsformate aber auch ernst nehmen und zudem bereit sein, einen Beitrag zur Sicherung von Frieden und Stabilität in unseren Nachbarregionen und in der Welt zu leisten. Zu lange haben wir uns auf die USA verlassen, die diese Verantwortung über Jahrzehnte geschultert haben. Für dieses Ungleichgewicht schwindet in der US-amerikanischen Gesellschaft der Rückhalt – und zwar entlang des gesamten politischen Spektrums, auch bei den Demokraten. Außerdem sollten wir den Dialog mit den Vereinigten Staaten auf allen Ebenen intensivieren, die transatlantischen Handelsbeziehungen vertiefen, die europäische Handlungsfähigkeit sowie den Zusammenhalt der NATO stärken und so dazu beitragen, die besonderen Beziehungen zwischen Europa und den USA auf eine Grundlage zu stellen, die den aktuellen geopolitischen Umbrüchen Rechnung trägt.

### Klare Haltung gegenüber Russland – Gesprächskanäle offenhalten, Werte verteidigen

Russland hat durch die völkerrechtswidrige Annexion der Krim im Jahr 2014 und seine – nicht offiziell eingeräumte – Beteiligung am bewaffneten Konflikt in der Ostukraine die seit 1945 geltenden Prinzipien der europäischen Friedensordnung in Frage gestellt und die Prinzipien der KSZE^2^-Schlussakte, der Charta von Paris und des Budapester Memorandums missachtet. Auch im Inland hat die russische Regierung durch ihre zunehmend repressive Politik gesellschaftliche und wirtschaftliche Modernisierung sowie fairen demokratischen Wettbewerb unterdrückt. Sie hat die Arbeit der Oppositionsparteien nahezu unmöglich gemacht und die dringend notwendige Diversifizierung der Wirtschaft verpasst. Für uns Freie Demokraten sind die Prinzipien des Völkerrechts, der Menschenrechte und der europäischen Friedensordnung, zu denen sich auch Russland bekannt hat, nicht verhandelbar. Wir setzen uns daher für ein unverzügliches Ende der Gewalt in der Ostukraine und der völkerrechtswidrigen Annexion der Krim ein und stehen ausdrücklich zu den von der EU verhängten Sanktionen. Im Fall einer weiteren militärischen Eskalation in der Ukraine muss die EU diese verschärfen, denn sie sind kein Selbstzweck, sondern dienen der Wiederherstellung der Friedensordnung. Bei einem substanziellen Einlenken Russlands können sie deshalb auch gelockert oder aufgehoben werden. Insgesamt sehen wir die derzeitige Politik der Russischen Föderation mit großer Sorge. Die russische Unterstützung für den syrischen Diktator Baschar al-Assad oder den libyschen Rebellenführer Chalifa Haftar sind ebenso kontraproduktiv für engere Beziehungen mit der EU wie wiederholte Desinformationskampagnen und Hackerangriffe in Europa oder der gezielte Giftanschlag auf den russischen Oppositionspolitiker Alexej Nawalny. Schon der langjährige FDP-Außenminister Hans-Dietrich Genscher hat immer betont, dass Europa nicht an der Außengrenze der EU endet. Besonders Russland bleibt uns menschlich, kulturell und wirtschaftlich eng verbunden. Auf Basis dieser Verbindungen müssen Deutschland und die EU Gesprächskanäle, insbesondere zur Zivilgesellschaft, offenhalten. Auch sollten Reisen für die jungen Menschen durch Visumserleichterung erleichtert werden. Die russische Regierung muss zuvor aber zu Rechtsstaatlichkeit sowie der Einhaltung der Bürgerrechte und des Völkerrechts zurückkehren. Nur so kann wieder das Vertrauen entstehen, das notwendig ist, um bei internationalen Problemen konstruktiv zusammenzuarbeiten.

### Klarheit in den China-Beziehungen

Die chinesischen Repressionen gegenüber den Aktivist*innen der Freiheitsbewegung von Hongkong, Pekings Drohgebärden gegenüber Indien oder die aggressive Einschüchterungspolitik gegenüber Taiwan haben im letzten Jahr ebenso die Schlagzeilen beherrscht wie der Handelskonflikt zwischen den USA und China. Die Meldungen zeigen beispielhaft, wie der Aufstieg Chinas die internationale Ordnung verändert und die westlichen Staaten wirtschaftlich, gesellschaftlich und geopolitisch herausfordert. Viel wird deshalb davon abhängen, wie es der internationalen Gemeinschaft gelingt, die tektonische Machtverschiebung durch den chinesischen Aufstieg zu gestalten. Deutschland und die EU müssen sich auf diese Veränderungen vorbereiten. Dabei ist eine Zusammenarbeit mit China auf Augenhöhe nur im europäischen Schulterschluss möglich. Ein engerer Austausch mit China kann nur auf der Grundlage und der Einhaltung des geltenden internationalen Rechts und insbesondere der Regeln der Vereinten Nationen und der Welthandelsorganisation erfolgen. Das erwarten auch und gerade Chinas Nachbarn von der EU, die oftmals von China eingeschüchtert werden. Kooperation mit Peking darf nicht erkauft werden, indem Menschenrechtsverletzungen oder fehlende Rechtsstaatlichkeit verschwiegen werden. Gegenseitiger Marktzugang und die universelle Geltung der Menschenrechte sind untrennbarer Teil unseres multilateralen Werte- und Regelsystems. Die beispiellose technische Überwachung der Bevölkerung sowie Unterdrückung und Internierung ethnischer und religiöser Minderheiten durch den chinesischen Staat und die Internierung und Zwangssterilisierung muss im Rahmen des EU-China-Dialogs mit Nachdruck thematisiert werden.

Gleichzeitig muss die EU an der Seite der mutigen Bürger*innen von Hongkong stehen, die für ihre Freiheitsrechte eintreten. Wir beobachten mit großer Sorge, wie das neue Sicherheitsgesetz der chinesischen Führung die demokratischen Proteste in Hongkong noch stärker kriminalisiert, unterdrückt und Oppositionspolitiker*innen an der Ausübung ihrer Mandate hindert. Zusammen mit der EU und den USA muss Deutschland die Einhaltung der chinesisch-britischen Erklärung von 1984 einfordern, die das Prinzip „Ein Land, zwei Systeme“ verankert. Gleichzeitig muss Europa den Blick auf Asien weiten und die Beziehungen zu solchen Staaten vertiefen, die anders als China Wertepartner sind. Besonders Japan, Australien, Indien und die ASEAN-Staaten gehören stärker in den Fokus deutscher und europäischer Außenpolitik.

## Krisen erfordern aktive Diplomatie – Herausforderungen für die deutsche Außenpolitik

### Für eine Wiederbelebung des Friedensprozesses im Nahen Osten

Israel und Deutschland sind in engster Freundschaft verbunden. Diese Freundschaft und das Vertrauen zwischen beiden Staaten und ihren Bürger*innen sind ein historischer Glücksfall, der nach dem Zivilisationsbruch der Shoah kaum mehr möglich erschien. Deutschland trägt deshalb eine besondere Verantwortung gegenüber Israel. Die Sicherheit und das Existenzrecht Israels als jüdischer und demokratischer Staat sind und bleiben deutsche Staatsräson und damit ein Grundpfeiler unserer liberalen Außen- und Sicherheitspolitik. Die Sicherheit Israels als einzige Demokratie im Nahen Osten ist aber nahezu untrennbar mit der Stabilität und dem Frieden in der Region insgesamt verbunden. Deswegen begrüßen wir grundsätzlich die positiven Entwicklungen im israelisch-arabischen Normalisierungsprozess. Die diplomatischen Bemühungen um Lösungen im israelisch-palästinensischen Konflikt konnten in den vergangenen Jahren keinerlei relevante Erfolge erzielen. In der internationalen Gemeinschaft herrscht dennoch weiterhin Einigkeit darüber, dass der israelisch-palästinensische Konflikt nur im Wege einer sogenannten Zweistaatenlösung, zu der sich die Konfliktparteien im Abkommen von Oslo 1993 bekannt haben, und unter Achtung des Völkerrechts friedlich beigelegt werden kann. Die Zweistaatenlösung zeigt bislang den einzigen Weg auf, um einerseits dauerhaft die Sicherheit des jüdischen und demokratischen Staates Israels zu garantieren und andererseits die Schaffung eines souveränen, demokratischen und lebensfähigen Staates Palästina zu ermöglichen. Deutschland sollte deshalb gemeinsam mit den europäischen Partnern im Dialog mit den USA, den Vereinten Nationen und relevanten Akteuren in der Region, wie Saudi-Arabien, Jordanien und Ägypten, mit Nachdruck für neue Verhandlungen zwischen Israel und Palästina werben.

### Nukleare Bewaffnung des Iran verhindern

Der Fortbestand des Nuklearabkommens mit dem Iran (Joint Comprehensive Plan of Action, JCPoA) ist eine der großen Herausforderungen für die internationale Diplomatie im Jahr 2021. Der JCPoA markiert nicht nur eine entscheidende Wegmarke für die nuklearen Nichtverbreitungsbemühungen im Nahen Osten, sondern auch einen Erfolg europäischer Diplomatie, insbesondere des EAD. Seit dem Rückzug der USA unter Trump im Jahr 2018 erodiert das JCPoA immer stärker. Immer mehr iranische Verstöße gegen die Verpflichtungen des Landes aus dem JCPoA machen es immer schwieriger, die Vereinbarung am Leben zu erhalten. US-Präsident Biden hat bereits seine Bereitschaft erklärt, dem JCPoA wieder beizutreten, sollte Iran zur Einhaltung der Vereinbarungen des Nuklearabkommens zurückzukehren. Als Freie Demokraten begrüßen und unterstützen wir die Bemühungen der neuen US-Administration, sich gemeinsam mit den EU‑3 für die Rettung des JCPoA einzusetzen. Auch ist es richtig, Gespräche mit Iran über die dauerhafte Verlängerung der Beschränkungen aus dem JCPoA und sein ballistisches Raketenprogramm aufzunehmen. Oberstes Ziel der Nichtverbreitungsbemühungen im Nahen Osten bleibt es, eine nukleare Bewaffnung des Iran dauerhaft zu verhindern, denn der Iran nimmt unbestritten eine destabilisierende Rolle in der Region ein. Die iranische Führung ist eine der wichtigsten Unterstützer des syrischen Regimes unter Assad und der Aktivitäten der libanesischen Hisbollah. Iran spielt eine aktive Rolle in den Konflikten in Syrien und im Jemen. Auch versucht Iran konsequent, seinen Einfluss im Nachbarstaat Irak auszubauen. Insgesamt unterstreicht Iran mit seinen Aktivitäten die Ambitionen des Landes als Regionalmacht. Die Nachbarstaaten des Iran und die internationale Gemeinschaft beobachten diese Entwicklung mit äußerster Sorge. Insbesondere die israelfeindliche Politik des Iran verurteilen wir mit Nachdruck.

### Beziehungen zur Türkei auf eine neue Grundlage stellen

Die Beziehungen zur Türkei sind für die Bundesrepublik Deutschland von besonderer Bedeutung, nicht nur aufgrund der ca. drei Millionen Menschen in Deutschland mit türkischen Wurzeln. Die Türkei ist NATO-Partner und seit 1999 EU-Beitrittskandidat, es gibt zahlreiche politische Kooperationsformate wie den strategischen Dialog auf Außenministerebene oder die zweijährlichen bilateralen Regierungskonsultationen. Auch der wirtschaftliche Austausch ist sehr intensiv. Gleichzeitig hat der türkische Präsident Recep Tayyip Erdoğan die Türkei in den letzten Jahren mehr und mehr zu einem autoritären Staat umgebaut und insbesondere die Presse- und Versammlungsfreiheit sowie die Unabhängigkeit der Justiz massiv eingeschränkt. Der türkische Staat schreckt längst nicht mehr vor der Verhaftung ausländischer Journalist*innen oder einheimischer Oppositionspolitiker*innen und Aktivist*innen zurück. Der autoritäre Umbau des türkischen Staates begann nicht erst mit dem gewaltsamen Putschversuch von 2016, gewann aber danach eine neue Qualität. Die jüngste Ernennung eines loyalen Vertreters der Partei für Gerechtigkeit und Entwicklung (Adalet ve Kalkınma Partisi, AKP) als neuen Rektor der renommierten Boğaziçi Universität in Istanbul reiht sich nahtlos ein in den repressiven Kurs Erdoğans und die zunehmende Gleichschaltung von Medien, Wissenschaft und Kultur sowie die Überwachung von Anwält*innen, Menschenrechtler*innen und Oppositionell*innen. Auch außenpolitisch verfolgt die Türkei unter Präsident Erdoğan einen immer aggressiveren Kurs. Die Türkei ist nicht nur im libyschen Bürgerkrieg, sondern auch in Nordsyrien, im Irak und jüngst in Berg-Karabach militärisch aktiv. Zudem eskalierte Ankara im Jahr 2020 den Streit mit EU-Mitglied und NATO-Partner Griechenland über Hoheitsgebiete im östlichen Mittelmeer. Positiv anzumerken ist, dass die Türkei im Januar 2021 einen bewusst diplomatischeren Kurs eingeschlagen und deutliche Signale der Gesprächsbereitschaft an Griechenland, Zypern und die EU gesandt hat.

Dennoch hat das letzte Jahr deutlich gezeigt, dass die Türkei sich immer weiter von der EU und ihrem Wertefundament entfernt hat. Im letzten Fortschrittsbericht vom Oktober 2020 hat die EU-Kommission der Türkei erneut ein schlechtes Zeugnis ausgestellt und weitere ernsthafte Rückschritte mit Blick auf Demokratie, Rechtsstaatlichkeit, Grundrechte und die Unabhängigkeit der Justiz festgestellt. Wir Freie Demokraten erkennen die besondere Rolle der Beziehungen zwischen Deutschland und der EU mit der Türkei. Sowohl die geographische Lage der Türkei als auch die große türkische Community in Europa verpflichten uns zu einem Neuanfang im Verhältnis zur Türkei. Wir wollen deshalb die Beitrittsverhandlungen der EU mit der Türkei in der bisherigen Form beenden und die Beziehungen mit der Türkei auf eine neue Grundlage enger sicherheitspolitischer und wirtschaftlicher Zusammenarbeit stellen. Die Türkei ist und bleibt als NATO-Mitglied und Nachbar ein unverzichtbarer Partner, weswegen es wichtig ist, die sicherheitspolitischen Spannungen im Bündnis abzubauen.

### Neue Impulse für Frieden in der Ukraine

2021 jährt sich die völkerrechtswidrige Annexion der Krim durch Russland zum siebten Mal. Nachdem bewaffnete Truppen ohne Hoheitsszeichen Verwaltungsgebäude und Infrastrukturanlagen auf der ukrainischen Halbinsel blockierten, bewilligte die russische Duma am 1. März 2014 Streitkräfte auf dem Territorium der Ukraine. Seither führt Russland in den Regionen Donezk und Luhansk im Osten der Ukraine einen hybriden Krieg, in dem nach Schätzung der Vereinten Nationen bislang über 13.000 Menschen starben.^3^ Seit fast sieben Jahren versucht die EU unter deutsch-französischer Federführung, den Konflikt in der Ostukraine diplomatisch zu lösen. Auch wenn die im Juli 2020 vereinbarte Waffenruhe der Trilateralen Kontaktgruppe, bestehend aus der Ukraine, Russland und der Organisation für Sicherheit und Zusammenarbeit in Europa (OSZE), nach vielen erfolglosen Anläufen derzeit weitgehend hält, bleiben weitere Erfolge zur nachhaltigen Friedenssicherung aus. Die internationale Gemeinschaft sollte deshalb in 2021 eine neue diplomatische Initiative anstoßen, um einen dauerhaften Frieden in der Ukraine zu erreichen. Wir setzen uns dafür ein, dass Deutschland auf EU-Ebene und in direkten Verhandlungen mit Russland und der Ukraine im Normandie-Format auf einen ergänzenden Aktionsplan zum bereits vereinbarten Maßnahmenpaket hinarbeitet. Dieser Aktionsplan sollte Maßnahmen priorisieren, eine schrittweise Umsetzung sicherheitsrelevanter und politischer Punkte vorsehen und mit verbindlichen Fristen sowie konkreten Konsequenzen bei (Nicht‑)Umsetzung Anreize zur Einhaltung schaffen und auf Basis des von der OSZE vorgeschlagenen Diskussionspapiers „Joint United Nations/OSCE Mission to Eastern Ukraine“ im UN-Sicherheitsrat einen umfassenden Friedensplan für die Ukraine erarbeiten und umsetzen.

## Footnotes

1 Csaky, Z. (o. J.). Nations in transit 2020. Dropping the democratic facade. Freedom House. https://freedomhouse.org/sites/default/files/2020-04/05062020_FH_NIT2020_vfinal.pdf. Zugegriffen: 29. März 2021.

2 Konferenz über Sicherheit und Zusammenarbeit in Europa.

3 Vereinte Nationen (2019, 16. Juli). Human cost of Ukraine conflict in growing, Security Council told. https://news.un.org/en/story/2019/07/1042561. Zugegriffen: 29. März 2021.

